# Treatment of striae albae with 1,550 nm Er: Glass vs. CO_2_ fractional laser: A self-controlled study

**DOI:** 10.3389/fmed.2022.1060815

**Published:** 2023-01-10

**Authors:** Yanfei Luo, Yan Lin, Meiling Wang, Xiaoyuan Gao, Xiaodong Liu, Huaxu Liu

**Affiliations:** ^1^Department of Dermatology, Hainan General Hospital, Haikou, Hainan, China; ^2^Shandong Provincial Hospital for Skin Diseases, Shandong First Medical University & Shandong Academy of Medical Sciences, Jinan, Shandong, China; ^3^Department of Plastic Surgery, Third People’s Hospital of Guizhou, Guiyang, Guizhou, China

**Keywords:** 10,600 nm CO_2_ fractional laser, striae albae, comparative study, non-ablative 1,550-nm Er: glass fractional laser, abdomen

## Abstract

**Objective:**

The objective of this study was to compare the efficacy and safety of fractional CO_2_ laser and 1,550 nm Er: glass laser in the treatment for the patients with striae albae.

**Methods:**

The female adults with striae albae in the abdomen for at least 12 months were recruited. After informed consent obtained, the patient received three treatments at 2-month intervals. The lesions on the left abdomen were treated with 10,600 nm CO_2_ fractional laser and right side treated with 1,550 nm Er: glass fractional laser. The pictures were taken before each visit and 3 months after the final treatment. The criteria for the evaluations using a quartile grading scale were excellent (76–100%), good (51–75%), fair (26–50%), poor (1-25%), and no improvement (0%). The safety and efficacy of the two lasers were independently evaluated using before and after photographs by two dermatologists. In addition, the self-reports to investigate the pain and satisfaction from patients were also recorded.

**Results:**

Totally, 27 cases were recruited, and 25 patients completed the treatments and follow-up. The excellent and good results (improvement of 51–100%) were achieved on the right abdomen in 84% of the patients, while 48% on the left site (*p* < 0.05). Hyper-pigmentation was seen in 20% of the patients assessed on the left abdomen and only in 8% on the right abdomen. During the treatments, average score of the pain on the right abdomen was 5.41 ± 2.13, which was higher than that on the left (4.19 ± 2.12) (*p* < 0.001). No permanent hyper-pigmentation was found on the both sides. Considering the whole treatments, the patients favored the modality used on the right side (80 vs. 68%, *p* < 0.05).

**Conclusion:**

Compared with CO_2_ fractional laser, 1,550 nm Er: glass fractional laser therapy provides the significantly better clinical outcomes and fewer side effects in the treatment of striae albae.

**Limitations:**

The sample size and follow-up time were limited.

## Introduction

Striae distensae (SD) or stretch marks are common and disfiguring dermatologic conditions, which usually arise due to obesity, pregnancy, rapid weight gain or weight loss, certain endocrine conditions, and prolonged use of steroids (1–5). Initially, typical lesions on the abdomen, breasts, thighs, or buttocks present as raised edematous linear plaques or stretched flat (striae rubrae), after which the color fades and turns white and atrophic (striae albae) as a result of local breakdown and reorganization of collagen and elastin bundles (3–6).

According to the domestic and international literature reports, the incidence of stretch marks is approximately 60–90%, and most of them begin to appear around 24 weeks of pregnancy (7, 8). The SD, especially striae albae present with permanent lesions, increasingly cause considerable psycho-social distress, which often leads to a decrease in quality of life (3, 4, 6). The substantial demand for a reliable treatment option is increasing to improve the persistent SD lesions in recent years; there are many treatment modalities used to treat SD, including topical agents, such as tretinoin ointment and olive oil, microneedles, and the energy-based devices, such as RF, pulsed laser, and fractional lasers (9–14).

There is no golden standard in the treatment of SD until now. As reported previously (15–20), both ablative and non-ablative fractional lasers showed certain clinical efficacy in the treatment of striae albae, but most of the studies were clinically retrospective study, without strict design and control. The well-designed controlled study is in urgent need to investigate the efficacy and side effects in the treatment of striae albae with different modalities.

The purpose of our study was to evaluate and compare the clinical efficacy and safety of 10,600 nm ablative CO_2_ and 1,550 nm non-ablative Er: glass fractional lasers for the treatment of striae albae by self-controlled studies.

## Patients and methods

### Patient preparation

This study was a prospective self-controlled clinical study and approved by the Ethics Committee of Shandong Provincial Hospital for Skin Diseases. Female patients with striae albae in the abdomen were enrolled in the study.

#### Inclusion criteria

The inclusion criteria were as follows: bilateral symmetrical striae albae without any treatments in the abdomen and stable for more than 1 year.

#### Exclusion criteria

The exclusion criteria were as follows: pregnant or lactating women; patients with a history of psoriasis, vitiligo, or keloid; suffering from light-sensitive system diseases, such as systemic lupus erythematosus, dermatomyositis; people with coagulopathy; scarred body; patients with active infection at the treatment site; recent abdominal glucocorticoid ointment; recent retinoid drugs; receive other physical treatments within 1 year; and patients with high or unfavorable expectations.

After written informed consent obtained, the patients received pictures taken before the treatment. Then, the whole abdomen lesions were covered for 1 h after a topical application of compound lidocaine cream (Beijing Ziguang Pharmaceutical Co., Ltd., with 25 mg of lidocaine per gram and 25 mg of prilocaine per gram). Then 1 h later, the lesions were cleaned and disinfected with iodophor and normal saline.

### Laser therapy

Treatment systems used in our study were 10,600 nm CO_2_ fractional laser (Queen, WON TECH Co., Ltd., Korea) and 1,550 nm Er: glass fractional laser (sellas-evo, Dinoa Inc., Korea).

The striae treatment areas were divided into halves using the patients’ umbilicus as the middle line on the abdomen. The left side is treated with 10,600 nm CO_2_ laser and the laser settings are similar, with 10,600 nm wavelength, 60–75 mJ energy, 1.5-mm point spacing, and 20 mm × 20 mm scanning area. On the right side, a 1,550 nm Er: glass fractional laser treatment was used. The treatment parameters were as follows: DD mode, spot density: 49 spots/cm^2^, energy density: 60–80 mJ, scanning area: 20 mm × 20 mm. The laser settings used in this study were determined based on our previous experience with these lasers and the therapeutic response of the patients.

The pulsed were not overlapped during treatment and the lesions were cooled with an ice pack for 10–15 min immediately after the treatment, and then, fusidic acid ointment was used two times daily for 3 days.

All the patients received three times laser treatments at 2-month intervals and were followed by 3 months after the final treatment.

### Evaluation

Before each treatment and 3 months after the final treatment, standard photographs were taken using a digital camera (Nikon D7000, Japan) under the same conditions, and two independent clinicians evaluated the pictures.

The criteria for the evaluations using a quartile grading scale were as follows: 0 = no improvement, 1 = poor (1-25%), 2 = fair (26–50%), 3 = good (51–75%), and 4 = excellent (76–100%) (15).

The patients’ self-satisfaction and pain scores were also recorded after the treatments. The patients’ self-satisfaction was divided into very satisfied (improved 76–100%), satisfied (51–75%), generally satisfied (26–50%), and dissatisfaction (0–25%) (16). The two-sided pain was recorded during the treatment and visual scoring was used with 0 points = no pain and 10 points = extremely painful.

After each treatment, the condition of disappearance of erythema, edema, peeling off time, hyper-pigmentation, and hypo-pigmentation were recorded by the questionnaire after treatment.

### Statistical analysis

All statistical analyses were carried out using SPSS Statistics 20.0 (IBM, Armonk, New York, USA). Data were expressed as mean ± standard deviation and statistical significance was accepted for *p*-values of less than 0.05. Statistical analysis method was tested using Wilcox rank sum test.

## Results

Totally, 27 cases were treated and two cases were lost during follow-up. Totally, 25 patients with striae albae were enrolled in in the final analysis (of which 20 were Fitzpatrick skin type III and five were IV type). The patients ranged in age from 26 to 38 years (mean 32.93 ± 3.71 years), and the duration of striae albae ranged from 1 to 13 years (mean 5.12 ± 2.95 years).

**FIGURE 1:**
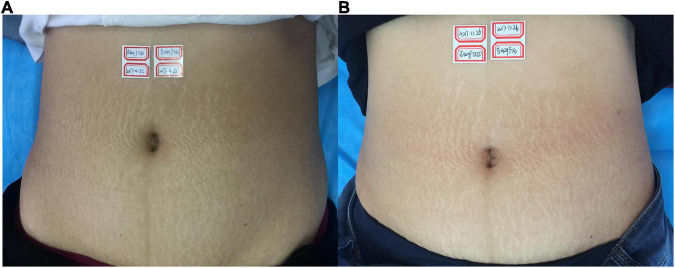
**(A)** Before treatment; **(B)** 3 months after the final treatment, the left clinical efficacy score was 2; the right side was 3.

**FIGURE 2:**
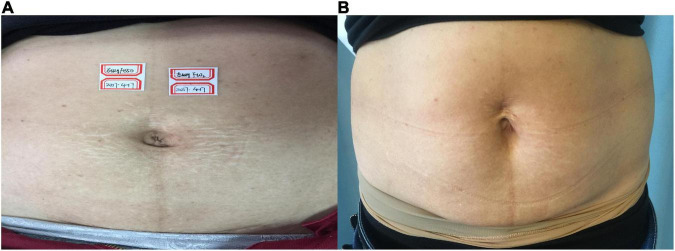
**(A)** Before treatment; **(B)** 3 months after the final treatment, the left clinical efficacy score was 3; the right side was 4.

**FIGURE 3:**
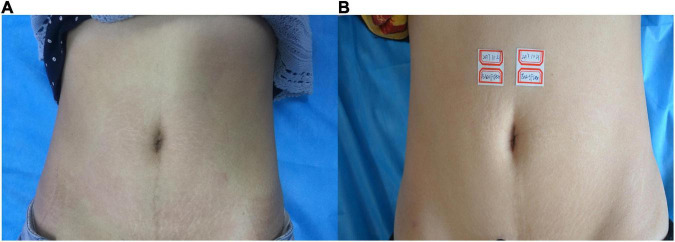
**(A)** Before treatment; **(B)** 3 months after the final treatment, the left clinical efficacy score was 3; the right side was 4.

**FIGURE 4:**
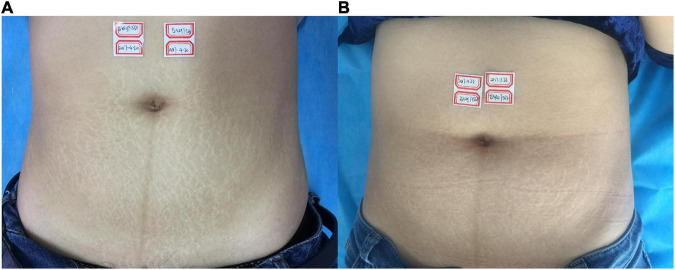
**(A)** Before treatment; **(B)** 3 months after the final treatment, the left clinical efficacy score was 3; the right side was 4.

**FIGURE 5:**
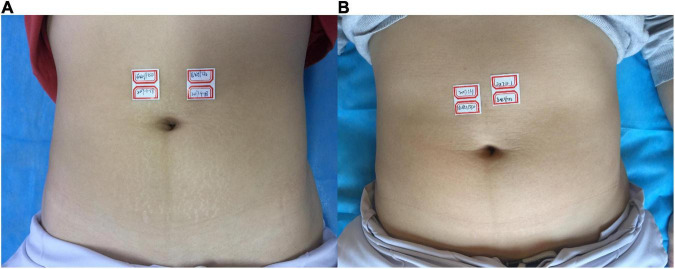
**(A)** Before treatment; **(B)** 3 months after the final treatment, the left clinical efficacy score was 4; the right side was 4.

There was no aggravation of stretch marks on both sides treated. The results of the two lasers are shown in Table 1 and Figures 1–5. There was a significant difference in clinical efficacy between the two sides (*p* = 0.002, *p* < 0.05) (Figures 1–5 and Table 1).

**TABLE 1:** Results of quartile improvement scale.

Quartile improvement scale	No. of patients (%)	
	CO_2_ FL	1,550 nm Er: glass FL
0 = No improvement	0 (0%)	0 (0%)
1 = Poor (1–25%)	0 (0%)	0 (0%)
2 = Fair (26–50%)	13 (52%)	4 (16%)
3 = Good (51–75%)	11 (44%)	13 (52%)
4 = Excellent (76–100%)	1 (4%)	8 (32%)
*P*-value	<0.05	

CO_2_ FL, fractional CO_2_ laser; 1,550 nm Er: glass FL, 1,550 nm Er: glass fractional laser.

The self-evaluation of patient satisfaction with striae albae improvement was similar to the picture assessments and is shown in Table 2. The difference in patient satisfaction between the two treatment methods was statistically significant (*p* = 0.034, *p* < 0.05) (Table 2).

**TABLE 2:** Likert satisfaction scale.

Likert satisfaction scale	No. of patients (%)	
	CO_2_ FL	1,550 nm Er: glass FL
1 = Very dissatisfied	0 (0%)	0 (0%)
2 = Dissatisfied	1 (4%)	0 (0%)
3 = Slightly satisfied	7 (28%)	5 (20%)
4 = Satisfied	16 (64%)	12 (48%)
5 = Very satisfied	1 (4%)	8 (32%)
*P*-value	<0.05	

CO_2_ FL, fractional CO_2_ laser; 1,550 nm Er: glass FL, 1,550 nm Er: glass fractional laser.

### Side effects

Immediately after treatment, mild housemasters’ edema occurred on both the sides. The time of erythema edema subsided by the left side (average 2.06 ± 1.47 days) remained shorter than the right side (average 2.22 ± 1.56 days) (*p* = 0.029, *p* < 0.05). Crusts in the right side remained longer (average 15.19 ± 7.81 days) than the left side (average 13.43 ± 7.26 days) (*p* < 0.001). When considering the pain evaluation, the 1,550 nm Er: glass fractional laser treatment (average 5.41 ± 2.13 scores) was considered more painful than CO_2_ fractional laser treatment (average 4.19 ± 2.12 scores). Post-inflammatory hyper-pigmentation was noted in two patients (8%) on the right side and in six patients (24%) on the left side.

## Discussion

The SD, especially the striae albae, is therapeutically challenging form of dermal scarring, which causes the significant psycho-social distress and the treatment requirement is increasing in recent years (17, 18). The fractional lasers may be a potential treatment modality for striae albae. The mechanism of fractional laser treatment is based on the theory of fractional photothermolysis (19, 20), and the factional damage of the epidermis and dermis by fractional laser results in the thermal effects promoting the synthesis of new collagen fibers, thereby improving the stretch marks. Gokalp et al. (20) reported 16 patients with SD treated by a 1,550 nm non-ablative fractional laser and followed up for 1 year after the last treatment, with a total effective rate of 81.25%. Another study confirmed that the efficacy of CO_2_ fractional laser is more effective than IPL in the treatment of SD (21). For the comparatively deeper effect by fractional CO_2_ laser than IPL.

In this study, we performed the comparative study to compare the ablative CO_2_ vs. non-ablative fractional 1,550 nm laser for the treatment of striae albae. The previous study showed that the ablative CO_2_ fractional laser may provide better efficacy than non-ablative 1,550 nm fractional laser (16), and the treatment interval in this study was only 4 weeks, which seems a bit short for the observation of collagen remodeling. Our study highlights that the 1,550 nm non-ablative fractional laser may have better efficacy in the treatment of striae albae by the blinded evaluation and the patients’ self-satisfaction report. The potential explanation may be due to the different laser settings used in the study. The energy of fractional CO_2_ laser was 60–75 mJ (the maximum energy of the system was 115 mJ),while the energy of NFL was 60–80 mJ (the maximum of 120 mJ), which was determined based on our previous experience with these lasers and the therapeutic response and tolerance of the patients. We found that when the fluence of NFL is increased, the effective penetration depth is also increased and more new collagen fiber synthesis and collagen remodeling in the dermis may be induced. Furthermore, when the fluence is high enough, the damage to the epidermis could also be observed. The concept of non-ablative and ablative comparatively depends on the laser settings. On the other hand, the higher energy of NFL with longer pulse duration leads to more pain, which is the main complaint of NFL in the treatment of striae albae.

The contents of melanin are relatively high in Asian skin, and the incidence of PIH after laser treatment is higher than that of Europeans and Americans (22). Of the 25 patients in our study, 20 were Fitzpatrick skin type III and five were type IV. The results of this study showed that the probabilities of PIH (24%) on the CO_2_ fractional laser treatment side were higher than those of 1,550 nm Er: glass fractional laser treatment side (8%), but neither of them had permanent pigmentation changes.

Our study found that the non-ablative fractional 1,550 nm laser may be more effective in the treatment of striae albae. However, the cases involved in the study and the follow-up time are limited. Further study to compare different laser settings for collagen remodeling with lasers is required to confirm our findings.

## Data availability statement

The raw data supporting the conclusions of this article will be made available by the authors, without undue reservation.

## Ethics statement

The studies involving human participants were reviewed and approved by Ethics Committee of Shandong Provincial Hospital for Skin Diseases. The patients/participants provided their written informed consent to participate in this study.

## Author contributions

HL designed the study. YFL, YL, and MW colleceted the enrolled cases. XG and XL followed the cases. YFL and HL prepared the manuscript. All authors contributed to the article and approved the submitted version.
